# SNP rs17079281 decreases lung cancer risk through creating an YY1-binding site to suppress DCBLD1 expression

**DOI:** 10.1038/s41388-020-1278-4

**Published:** 2020-03-30

**Authors:** Yu Wang, Rongna Ma, Ben Liu, Jinyu Kong, Hongyan Lin, Xiao Yu, Ruoyang Wang, Lei Li, Ming Gao, Baosen Zhou, Man Mohan, Herbert Yu, Zhaoyuan Hou, Hongbin Shen, Biyun Qian

**Affiliations:** 1 Hongqiao International Institute of Medicine, Shanghai Tongren Hospital and Faculty of Public Health, Shanghai Jiao Tong University School of Medicine, Shanghai, 200025 China; 2 Department of Cancer Epidemiology and Biostatistics, Tianjin Medical University Cancer Institute and Hospital, National Clinical Research Center of Cancer, Tianjin, 300060 China; 3 Department of Epidemiology, School of Public Health, China Medical university, Shenyang, Liaoning 110001 China; 4 Department of Biochemistry and Molecular Cell Biology, Shanghai Key Laboratory of Tumor Microenvironment and Inflammation, Shanghai Jiaotong University School of Medicine, Shanghai, 200025 China; 5 Cancer Epidemiology Program, University of Hawaii Cancer Center, Honolulu, HI 96813 USA; 6 Hongqiao Institute of Medicine, Shanghai Tongren Hospital/Faculty of Basic Medicine, Shanghai Jiaotong University School of Medicine, Shanghai, 200025 China; 7 Section of Clinical Epidemiology, Jiangsu Key Laboratory of Cancer Biomarkers, Prevention and Treatment, Cancer Center, Nanjing Medical University, Nanjing, 211166 China

**Keywords:** Predictive markers, Genetic predisposition to disease, Predictive markers, Genetic predisposition to disease

## Abstract

Genome-wide association studies (GWAS) have identified numerous genetic variants that are associated with lung cancer risk, but the biological mechanisms underlying these associations remain largely unknown. Here we investigated the functional relevance of a genetic region in 6q22.2 which was identified to be associated with lung cancer risk in our previous GWAS. We performed linkage disequilibrium (LD) analysis and bioinformatic prediction to screen functional SNPs linked to a tagSNP in 6q22.2 loci, followed by two case-control studies and a meta-analysis with 4403 cases and 5336 controls to identify if these functional SNPs were associated with lung cancer risk. A novel SNP rs17079281 in the *DCBLD1* promoter was identified to be associated with lung cancer risk in Chinese populations. Compared with those with C allele, patients with T allele had lower risk of adenocarcinoma (adjusted OR = 0.86; 95% CI: 0.80–0.92), but not squamous cell carcinoma (adjusted OR = 0.99; 95% CI: 0.91–1.10), and patients with the C/T or T/T genotype had lower levels of *DCBLD1* expression than those with C/C genotype in lung adenocarcinoma tissues. We performed functional assays to characterize its biological relevance. The results showed that the T allele of rs17079281 had higher binding affinity to transcription factor YY1 than the C allele, which suppressed DCBLD1 expression. DCBLD1 behaved like an oncogene, promoting tumor growth by influencing cell cycle progression. These findings suggest that the functional variant rs17079281C>T decreased lung adenocarcinoma risk by creating an YY1-binding site to suppress DCBLD1 expression, which may serve as a biomarker for assessing lung cancer susceptibility.

## Introduction

Lung cancer is the most common malignancies worldwide with 2.1 million new cases and 1.8 million deaths predicted in 2018 [1]. In China, the incidence of lung cancer has increased rapidly, posing significant social and economic challenges [2]. The development of lung cancer is known to be multifactorial. Genetic factors play an important role in disease susceptibility as well as in association with lung cancer risk factors, such as addiction to tobacco use. Although enormous efforts have been made to understand the etiology of lung cancer, the exact mechanisms of lung carcinogenesis remain to be elucidated.

The advent of GWAS (genome-wide association study) has provided a powerful avenue to investigate the genetic basis of disease susceptibility [3, 4]. GWAS examines common single nucleotide polymorphisms (SNPs) known as “tag SNPs” to identify their associations with disease risk [5]. Several GWAS reports have been published suggesting that a number of SNPs in chromosome 8q24, 3q28, 5q15.33, 3q12.12, 13q12.12, and 22q12.2 are associated with lung cancer risk in Han Chinese [6–10]. Since a large majority of the SNPs (~93%) lie within the nonprotein coding regions [11, 12], we face a huge challenge to understand the biological mechanisms underlying the associations between genetic polymorphisms and cancer risk. Thus, it is critical for us to identify the functional variants from those tag SNPs and characterize their biological functions related to tumorigenesis. Recently, the development of genome-editing technologies (CRISPR/Cas9) provides a new approach to assess the biological relevance of GWAS-identified SNPs to cancer [13].

In a previous GWAS [14], SNP rs9387478 in 6q22.2 was found to be associated with lung cancer risk in Asians, which has recently been reported for the same cancer in European populations [15]. SNP rs9387478 is located between two genes, *DCBLD1* and *ROS1*. *DCBLD1* encodes the discoidin, CUB and LCCL domain containing 1 protein (DCBLD1), and ROS1 is a proto-oncogene receptor tyrosine kinase. Both proteins are involved in regulation of cell proliferation and possibly invasion [16, 17]. In search for potential hidden causal SNPs in the 6q22.2 regions, we performed a linkage disequilibrium (LD) analysis to look for functional SNPs having high LD with rs9387478. For the SNPs with high LD, we analyzed their genotypes in association with lung cancer risk. In two case-control studies and a meta-analysis of 4403 cases and 5336 controls including additional three lung cancer GWASs data from our previous studies, we identified and confirmed that SNP rs17079281 was associated with lung cancer. We further performed functional experiments to demonstrate the biological implication of rs17079281 in lung cancer.

## Results

### Characteristics of study subjects

Demographic features and risk factors of lung cancer patients and their matched controls in our study are presented in Supplementary Table S1. Age and gender were not different between patients and controls, suggesting adequate matching on these factors. No significant differences were found between cases and controls in their history of lung diseases (*p* = 0.710) and family history of cancer (*p* = 0.072). As expected, smoking status was associated with the risk of lung cancer (OR = 1.66; 95% CI: 1.15–1.84; *p* < 0.001); a dose–response relationship was also noticed between the disease risk and pack-year of smoking. In addition, cases had lower BMI than controls (*p* < 0.001).

### DCBLD1 genotypes and lung cancer risk

A total of 39 SNPs were found in high LD with rs9387478, and of them four SNPs were selected to further analyze (Supplementary Table S2). Table 1 shows the genotypes of the *DCBLD1* SNPs in 766 cases and 773 controls and their associations with lung cancer after adjustment for age, gender, smoking status, BMI, and family history of cancer. Three SNPs (rs17079281, rs6911915, rs4946259) were in Hardy–Weinberg equilibrium (*p* > 0.01), and SNP rs9320604 was not. The distribution of SNP rs17079281 genotype was different between cases and controls, and the controls had more C/T heterozygous genotype than the patients (*p* = 0.021). Compared with individuals carrying the wild C/C genotype, those with C/T heterozygote had lower risk of lung cancer (adjusted OR = 0.74; 95% CI: 0.58–0.94). Under the dominant model, those with the T allele had an adjusted OR of 0.78 (95% CI: 0.63–0.98) compared with those with the C/C allele. No consistent associations with lung cancer were found for SNP rs4946259, rs9320604, and rs6911915.

**Table 1:** Associations between lung cancer and SNPs in high LD with rs9387478.

Genotype	*N* (%)		*P*^a^	OR (95% CI)^b^
	Control (773)	Case (766)		
*DCBLD1* rs17079281 (C>T)			**0.021**	
CC	338 (43.78)	375 (48.96)		1.00
CT	361 (46.76)	309 (40.34)		**0.74 (0.58–0.93)**
TT	73 (9.46)	82 (10.70)		1.06 (0.72–1.59)
* P* trend			0.246	
Dominant model				
CC	338 (43.78)	375 (48.96)		1.00
CT + TT	434 (56.23)	391 (51.04)		**0.78 (0.63–0.98)**
Recessive model				
CC + CT	699 (90.54)	684 (89.30)		1.00
TT	73 (9.46)	82 (10.70)		1.24 (0.85–1.80)
*DCBLD1* rs4946259 (G>A)			0.064	
GG	246 (31.82)	263 (34.33)		1.00
AG	408 (52.87)	366 (47.78)		0.79 (0.61–1.01)
AA	119 (15.39)	137 (17.89)		1.16 (0.82–1.62)
* P* trend			0.805	
Dominant model				
GG	246 (31.82)	263 (34.33)		1.00
AG + GG	527 (68.18)	503 (65.67)		0.87 (0.68-1.10)
Recessive model				
GG + AG	654 (84.60)	629 (82.11)		1.00
AA	119 (15.39)	137 (17.89)		1.33 (0.99-1.80)
*DCBLD1* rs6911915 (T>C)			0.090	
TT	219 (29.40)	244 (32.88)		1.00
CT	380 (51.01)	348 (46.90)		**0.77 (0.59–0.99)**
CC	146 (19.60)	150 (20.22)		0.92 (0.66–1.28)
* P* trend			0.447	
Dominant model				
TT	219 (29.40)	244 (32.88)		1.00
CT + CC	426 (57.18)	498 (67.12)		0.81 (0.63–1.03)
Recessive model				
TT + CT	599 (80.40)	592 (79.78)		1.00
CC	146 (19.60)	150 (20.22)		1.10 (0.83–1.44)
*DCBLD1* rs9320604 (G>A)			0.164	
GG	186 (28.18)	209 (32.20)		1.00
AG	410 (62.12)	381 (58.71)		0.79 (0.61–1.02)
AA	64 (9.70)	59 (9.09)		0.76 (0.50–1.17)
* P* trend			0.089	
Dominant model				
GG	186 (28.18)	209 (32.20)		1.00
AG + AA	474 (71.82)	440 (67.80)		0.78 (0.61–1.00)
Recessive model				
GG + AG	596 (90.30)	590 (90.91)		1.00
AA	64 (9.70)	59 (9.09)		0.89 (0.60–1.33)

^a^Two-side and calculated by logistic regression analysis.

^b^Adjusted by age, gender, smoke, and BMI.

Bold values indicates statistically significant values.

To validate our results on SNP rs17079281, we compared its genotypes in another case-control study with 558 cases and 534 controls. Table S3 shows that patients with the C/T genotype had lower risk of lung cancer compared to those with the C/C genotype (adjusted OR = 0.73; 95% CI: 0.56–0.95). A similar association was also observed in the dominant model. Patients with the T allele had an adjusted OR of 0.79 (95% CI: 0.62–1.00) compared to those with the C/C allele.

**Fig. 1:**
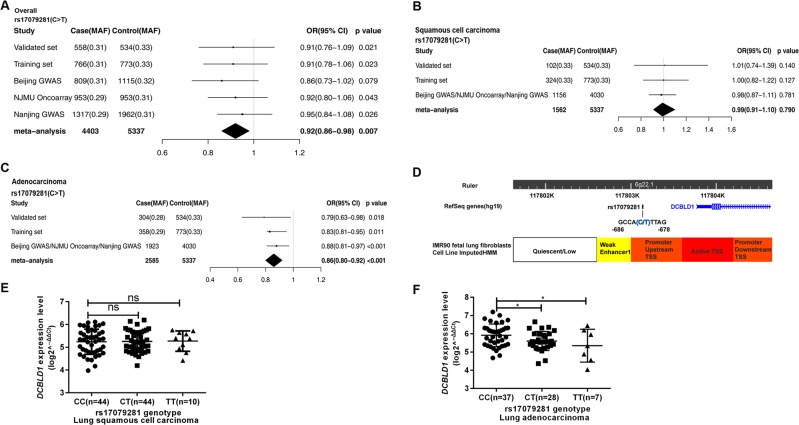
Forest plot represented the association between SNPrs17079281 and the risk of lung cancer overall and by histologic types and eQTL analysis. **a** All lung cancer based on 4403 cases and 5336 controls. The studies were weighted according to the proportioned to the sample size in each study. Combined ORs and 95%CI were derived from the per-allele model. The result for the fix effects model (*I*^2^ = 0%, *P* = 0.98) were presented. **b** Association between rs17079281 and risk of squamous cell carcinoma, **c** adenocarcinoma. **d** The relative positions of SNP rs17079281 and DCBLD1 gene mapping to 6q22.1 were shown. The chromatin state of 6q22.1 locus in IMR90 was detailed with chromatin state segmentation track (ChromhMM). **e**, **f** eQTL analysis demonstrating the correlation between rs17079281 genotype and expression of DCBLD1 in lung squamous cell carcinoma (**e**) and lung adenocarcinoma (**f**).

In addition, we performed meta-analysis on three additional GWAS studies, including a total of 4403 cases and 5336 controls. The result showed that patients with the T allele had an adjusted OR of 0.92 (95% CI: 0.86–0.96) compared to those with the C allele (Fig. 1a). Our findings were consistent among these studies. In analysis of association by histology, the SNP rs17079281 was associated only with adenomacarcinoma (adjusted OR = 0.86; 95% CI: 0.80–0.92) (Fig. 1c), but not squamous cell carcinoma (adjusted OR = 0.99; 95% CI: 0.91–1.10) (Fig. 1b).

### *DCBLD1* expression by rs17079281 genotypes in lung cancer tissue

SNP rs17079281 is located in the promoter of *DCBLD1*, which may affect the binding of transcription factors (Fig. 1d). To test whether the SNP could modulate *DCBLD1* expression, we measured *DCBLD1* mRNA expression in cancer tissue from lung cancer patients using quantitative PCR. We found that patients with homozygous T/T genotype or heterozygous C/T genotype had lower *DCBLD1* expression than those with C/C genotype in lung adenocarcinoma (Fig. 1e), but not in lung squamous cell carcinoma (Fig. 1f).

### Effect of rs17079281 on DCBLD1 transcription activity

**Fig. 2:**
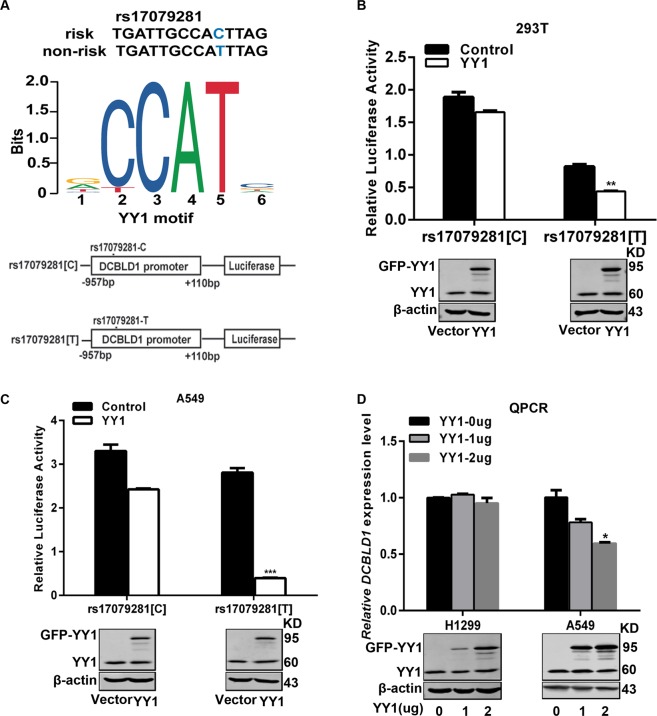
Luciferase expression assays with constructs containing *DCBLD1* promoter in different cell lines. **a** Bioinformatic analysis shows that C-to-T mutation creates a binding site for the transcription factor YY1 (Top). Schematic of the rs17079281 position relative to the transcription start site (TSS) and illustration of reporter constructs containing rs17079281 C or T allele (Bottom). **b**, **c** 0.4 μg of pGL3-rs17079281[C] (or pGL3-rs17079281[T] constructs cotransfected with 0.4 μg of vector or YY1 plasmid into 293T, A549, and 8 ng pRL-TK vector is cotransfected to standardize transfection efficiency. The experiments were carried out in triplicates in three independent transfection experiments. *P < 0.05; **P < 0.01, in comparison to pGL3-CC construct. Differences between groups were analyzed using two-side *t* test. **d** The levels of DCBLD1 as determined by RT-QPCR in lung cancer cell lines A549 (with C/T at rs17079281) and H1299 (with C/C at rs17079281) transiently transfected with 1 μg or 2 μg YY1 overexpression plasmid.

We used TRANSFAC software to predict that C>T mutation may create a transcription factor YY1 binding site (Fig. 2a, top). To assess if YY1 binds to the SNP region and the binding is affected by the SNP genotype, we constructed two luciferase reporter plasmids, one with C and one with T alleles of rs17079281 (Fig. 2a, bottom). Luciferase reporter assays showed that, in comparison to the construct with the rs17079281[C] allele, the construct with the rs17079281[T] allele had significantly reduced luciferase activity in the in 293T cells and lung cancer cells A549 with YY1 overexpression (Fig. 2b, c). In addition, overexpression of YY1 resulted in a significant decrease in expression of DCBLD1 in a dose–response manner in lung cancer cell line A549 (with C/T genotype at rs17079281), but overexpression YY1 in lung cancer cell line NCI-H1299 (with C/C genotype at rs17079281) did not have the same effect (Fig. 2d). These results support our speculation that rs17079281 regulated DCBLD1 expression by modulating the binding of transcriptional suppressor YY1 to its promoter.

### SNP rs17079281-dependent YY1 regulation of DCBLD1 expression

**Fig. 3:**
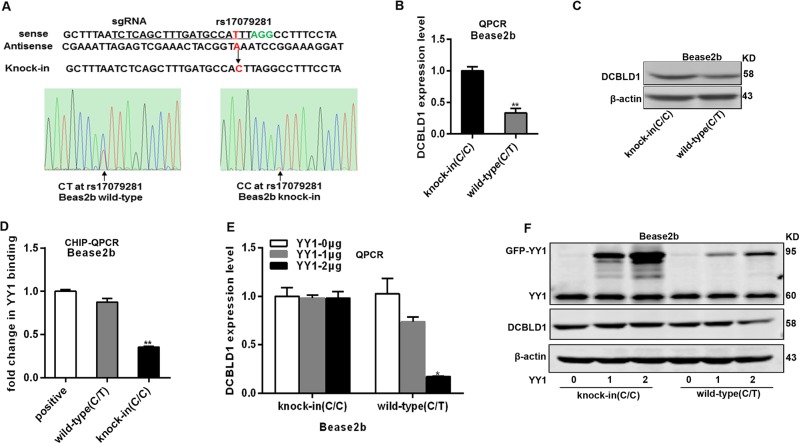
YY1-influenced DCBLD1 expression varied by rs17079281 genotype. **a** Top panels: schematic of homozygous knockin of rs17079281 C allele. The sgRNA protospacers are underlined, the SNP position is indicated in red and the PAMs in green. Bottom panels: the knockin homozygous(C/C) genotype clones were confirmed by Sanger sequencing. **b** The relative expression level of *DCBLD1* as determined by qRT-PCR in wild-type Beas2B cells (C/T) and the knockin clone (C/C). Results are shown as means ± SEM relative to β-actin levels and normalized to mean expression level in the knockin clone. The experiments are performed in three replicates. **P < 0.01 and *P* value is calculated with two-sided *t* tests. Immunoblot analysis of DCBLD1 protein in wild-type Beas2B cells(C/T) and the knockin clone (C/C). **d** CHIP assays are performed with control IgG or antibody to YY1 on the chromatin obtained from wild-type Beas2B cells (C/T) and the knockin clone (C/C), which are examined via qRT-PCR. Results are shown as means ± SEM and normalized to the positive control region from three independent experiments. **P < 0.01 and *P* value is calculated with two-sided *t* tests. **e**, **f** The expression level of DCBLD1 as determined by qRT-PCR (**e**) and western blotting (**f**) in wild-type Beas2B cells (C/T) and the knockin clone (C/C), which are transiently transfected with 1 μg or 2 μg of YY1 overexpression plasmid. Results are shown as means ± SEM relative to β-actin levels and normalized to control. The experiments are performed in three replicates. *P < 0.05 and *P* value is calculated with two-sided *t* tests.

To further demonstrate if rs17079281 C>T affects DCBLD1 expression, we used CRISPR/Cas9 to modify the genotype of rs17079281 in a normal lung cell line Beas2B (C/T at rs17079281). Using a T allele single-strand DNA oligonucleotide as a repair template, we obtained a homozygous C/C knockin clone at a frequency of 0.18% (1 out of 550), but no clone of homozygous T/T was detected (Fig. 3a). Compared with the homozygous C/C knockin clones, we observed a 50% decline in DCBLD1 expression in the cells with wild heterozygous (C/T) genotype (Fig. 3b, c). To examine if the YY1 binding affinity to *DCBLD1* differed between the C and T alleles of rs17079281, we performed CHIP-qPCR analysis on the wild-type Beas2B cells (C/T at rs17079281) and the modified cells with C/C knockin (Fig. 3d). The T allele of rs17079281 showed a higher binding affinity to YY1 than the C allele, supporting our speculation that the genetic polymorphism at rs17079281 confers an allele-specific binding ability to YY1. Further, when the Beas2B cells (C/T at rs17079281) were transfected with the YY1 overexpression plasmid, the DCBLD1 expression was markedly suppressed by YY1, and the suppression was dependent on the concentration of YY1 either at its mRNA or protein levels, but the C/C knockin cells did not show such effects (Fig. 3e, f).

### Increased lung cancer cell proliferation and cell cycle progression by DCBLD1

**Fig. 4:**
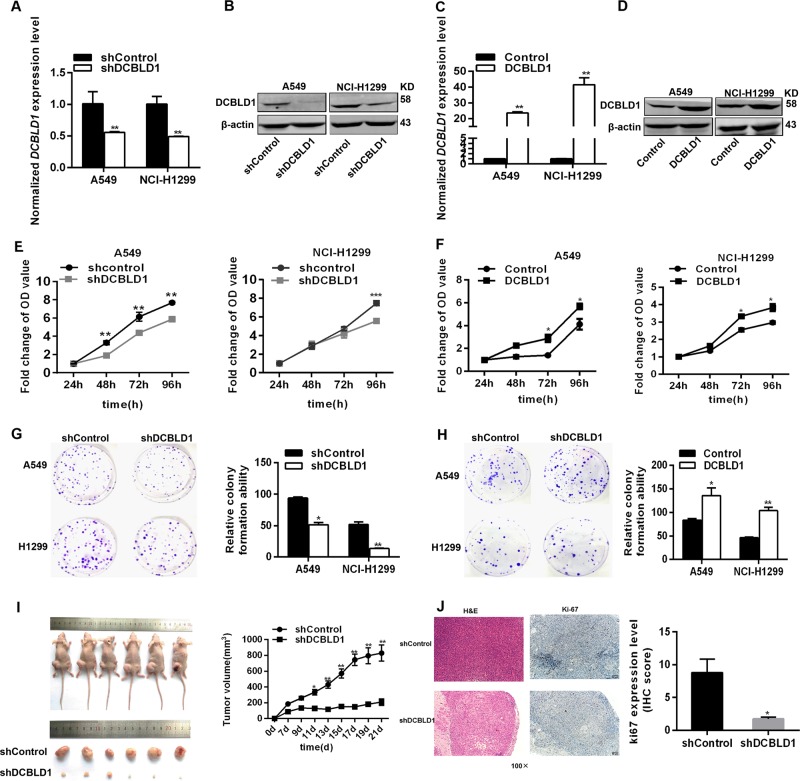
DCBLD1 expression promoted cell proliferation. **a**–**d** the expression of DCBLD1 was verified by qRT-PCR and western blot in the stable lung adenocarcinoma cells A549 and H1299 with knockdown (**a**, **b**) or overexpression of DCBLD1 (**c**, **d**). mRNA levels as determined by qRT-PCR are presented as means ± SE relative to shControl. **e** Knockdown of DCBLD1 substantially reduced the rate of cell proliferation in A549 and NCI-H1299, whereas overexpression of DCBLD1 substantially increased the rate of cell proliferation in A549 and NCI-H1299 (**f**). The stable cells with knockdown (**e**) or overexpression (**f**) of DCBLD1 were seeded in 96-well plates, and cell number was determined every 24 h for 96 h using CCK8 assays. Results are shown as the mean ± SEM from three experiments, each with six replicates. **P < 0.01 and ***P < 0.001, compared with control by two-sided *t* test. **g**, **h** Effect of DCBLD1 knockdown or overexpression on colony formation of lung cancer cells. The results presented colony formation ability relative to control cells. Results are shown as the mean ± SEM from three experiments, each with three replicates. *P < 0.05 and **P < 0.01, compared with control by two-sided *t* test. **i** Xenograft tumors in nude mice injected with A549 cells with stable DCBLD1 knockdown (left). Tumor growth curves were showed as means ± SEM, with six mice in each group (right). *P < 0.05; **P < 0.01; ***P < 0.001, compared with control by two-sided *t* test. **j** Portion of each xenograft tumor was fixed in 4% formaldehyde and embedded in paraffin. Tumor tissues were processed with H&E staining (Left) or specific Ki-67 antibody (Right) under 100× power microscope. *P < 0.05, compared with control by two-sided *t* test.

**Fig. 5:**
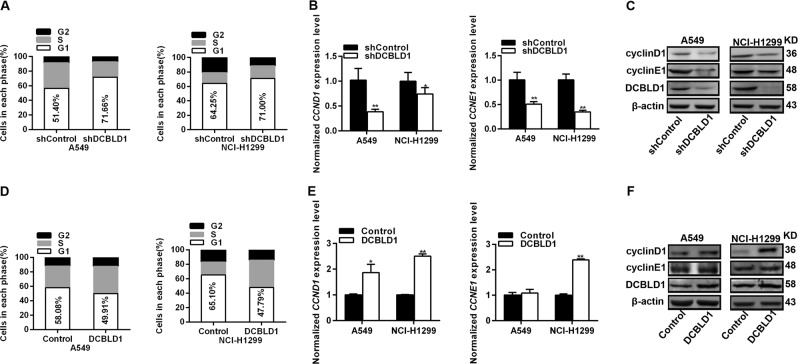
DCBLD1 expression promoted cell cycle progression. **a** Effect of DCBLD1 knockdown on cell cycle progression of lung cancer cells. **b** mRNA expression levels of *CCND1* and *CCNE1*in lung cancer cells with stably knockdown of DCBLD1. mRNA levels as determined by qRT-PCR were presented as means ± SE relative to shControl. **c** Immunoblot analysis of cyclinD and cyclinE in lung cancer cells with stably knockdown of DCBLD1. **d** Effect of DCBLD1 overexpression on cell cycle progression of lung cancer cells. **e** mRNA expression levels of *CCND1* and *CCNE1* in lung cancer cells with stably overexpression of DCBLD1. mRNA levels as determined by qRT-PCR were presented as means ± SE relative to shControl. **f** Immunoblot analysis of cyclinD and cyclinE in lung cancer cells with stably overexpression of DCBLD1. *p < 0.05; **P < 0.01, all *P* values are from two-sided *t* tests.

To examine the effects of DCBLD1 on lung adenocarcinoma cell behaviors, we established stable cells with knockdown or overexpression of DCBLD1. Our experiments showed that downregulation of DCBLD1 expression (Fig. 4a, b) in A549 and NCI-H1299 substantially reduced the rate of cell proliferation (Fig. 4e), whereas overexpression of DCBLD1 (Fig. 4c, d) increased cell proliferation (Fig. 4f). Colony formation of A549 and NCI-H1299 cells was suppressed by DCBLD1 knockdown and promoted by DCBLD1 overexpression (Fig. 4g, h). To evaluate if DCBLD1 had similar effects in vivo, we developed a xenograft model by injecting A549 cells with stable knockdown of DCBLD1 into nude mice (Fig. 4i). The tumor growth was significantly reduced after suppressing the expression of DCBLD1 (Fig. 4i) and cell proliferation marker Ki-67 was significantly decreased after suppressing the expression of DCBLD1 (Fig. 4j). Flow cytometry analysis suggested that DCBLD1 knockdown resulted in significant accumulation of A549 and NCI-H1299 cells in G1 phase, accompanied by a substantial decrease in S and G2 phases (Fig. 5a). In contrast, upregulation of DCBLD1 expression resulted in a decrease in G1 phase and increase in S phase (Fig. 5d). We found that expression of cyclin D1 and cyclin E1 in A549 and NCI-H1299 cells were downregulated by DCBLD1 knockdown (Fig. 5b, c) and upregulated with DCBLD1 overexpression (Fig. 5e, f).

## Discussion

**Fig. 6:**
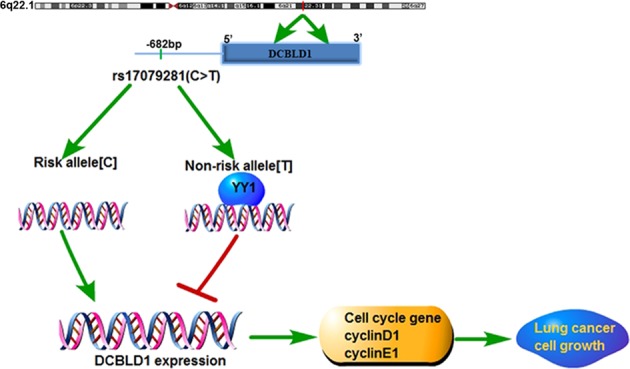
Functional action of DCBLD1 in modulating lung cancer risk. The C>T variation at SNPrs17079281 creating a YY1-binding site diminished DCBLD1 expression, which inhibit lung adenocarcinoma cell proliferation and influence the susceptibility to lung cancer.

We performed LD analysis on SNP rs9387478 discovered by GWAS in search for functional variants, and found that SNP rs17079281, located in the *DCBLD1* promoter, was associated with lung cancer. Intriguingly, we demonstrated that C>T transition at SNP rs17079281 created a YY1-binding site, resulting in decreased DCBLD1 expression. In addition, lower expression of DCBLD1 was associated with less cell proliferation, suggesting a role of oncogene in lung cancer development. These results indicated how a variant allele contributed to the susceptibility to lung cancer (Fig. 6).

Our genotype analysis showed that individuals with T allele at SNPrs17079281 had lower risk of lung cancer compared to those with the C allele. We further established that the SNP rs17079281 was distinctly associated with risk of Lung adenocarcinoma and not with the squamous cell carcinoma. SNP rs17079281 was located in the promoter region of the *DCBLD1* gene, 682 bp upstream from the transcription start site. Multiple studies have shown that SNPs in the regulatory regions alter gene expression in a tissue-specific manner [18]. To elucidate the effect of SNP rs17079281 on expression of gene *DCBLD1*, we analyzed the relationship between *DCBLD1* genotype and phenotype in lung tumor samples, and found an expression quantitative trait loci between SNP rs17079281 and *DCBLD1* mRNA levels in lung adenocarcinoma tissues, but not in lung squamous cell carcinoma. These findings suggest a histology-specific germ-line susceptibility to lung cancer risk, and more studies are warranted for further elucidation.

Bioinformatics analysis suggests that the C-to-T polymorphism is situated in a transcription factor YY1 binding site. In addition, it has been known that CCAT and ACAT are two types of core sequences that possess high binding affinity to YY1 in gene promoters [19]. Interestingly, the sequence surrounding the T allele of rs17079281 matches to the sequence of CCA**T**. YY1 has been implicated in the transcription repression of gene expression because YY1 possesses a strong repression domain at its C-terminus which consists of four GLI-Krüppel type zinc fingers and binds the CCAT sites [20]. The luciferase assay showed that transcription factor YY1 could suppress *DCBLD1* expression by interacting with the T alleles of rs17079281 more strongly than the C alleles in the *DCBLD1* promoter. Furthermore, in order to avoid the influence of confounding factors and to estimate the authentic association between SNP rs17079281 and *DCBLD1* mRNA levels, we used the CRISPR/cas9 technique to generate different genotypes in a normal lung cell line Beas2B which has a heterozygous (C/T) genotype at rs17079281. When the cells with heterozygous (C/T) genotype were transfected with the YY1 overexpression vector, the expression of DCBLD1 was decreased in a dose–response manner. Cells with the homozygous (C/C) genotype did not show the effect. Taken together, these finding indicated that transcription factor YY1 had a higher affinity to T allele which repressed DCBLD1 expression.

The *DCBLD1* gene is located at 6q22.2 which had been reported to be a susceptibility loci for colorectal tumors in a genome-wide meta-analysis [21]and an association with overall survival of small-cell lung cancer (SCLC) [22]. However, few studies have explored the biological function of DCBLD1 in lung cancer. Our study indicated that upregulation of DCBLD1 expression promoted lung cancer cell proliferation and downregulation of DCBLD1 diminished these effects in vitro. In vivo experiments also showed that downregulation of DCBLD1 inhibited tumor growth. In addition, significantly higher *DCBLD1* expression was found in lung cancer compared with adjacent normal tissue in a dataset available from TCGA (Supplementary Fig. S1). These results indicated DCBLD1 being an oncogene in lung cancer. SNP rs17079281 C>T variation created a site for YY1 binding which repressed the DCBLD1 expression, reducing its oncogenic effect on lung cancer development. Our mechanistic findings were consistent with the epidemiology results which showed that SNP rs17079281 T allele was associated with reduced risk of lung cancer.

In summary, in search for functional SNPs in high LD with SNP rs9387478 identified by lung cancer GWAS [14], we found that SNP rs17079281, located in the promoter region of *DCBLD1*, was associated with lung cancer risk. This SNP affected DCBLD1 expression through influencing its binding affinity to a transcription factor YY1. We demonstrated that *DCBLD1* promoted lung cancer cell proliferation by influencing cell cycle progression. However, as there is a low degree of orthology in the SNP site between human and animals, few in vivo animal model was avaliable for experiment. More studies are needed to further elucidate the biological mechanisms of DCBLD1 in tumorigenesis.

## Subjects and methods

### Study subjects

Associations between lung cancer and SNPs were analyzed in two groups of patients and controls, serving as training and validation sets. The training set included 766 cases and 773 controls recruited from Tianjin Medical University Cancer Hospital (TMUCH) between January 2006 and January 2013. The validation set consisted of 558 cases and 534 controls who were enrolled in another study at China Medical University. The two case-control studies were approved by the ethnic review committees at TMUCH and the institutional review board of China Medical University, respectively. Each study subject signed an informed consent before being enrolled in the study in accordance with the Declaration of Helsinki. The study participants also provided a 5 ml blood sample for research. We then did a meta-analysis of the two case-control studies and our previous lung cancer GWAS data (the Beijing GWAS with 809 cases and 1115 controls and Nanjing GWAS with 1317 cases and 1962 controls) [7] and unpublished lung cancer OncoArray data (the NJMU OncoArray with 953 cases and 953 controls). All patients in the study were diagnosed with histologically confirmed primary non-small cell lung cancer (NSCLC). Patients with a previous history of cancer or radio/chemotherapy were excluded from the study. Detailed information of study population was provided in Supplementary Methods.

### SNP selection and genotyping

Our search for causal SNPs was centered on SNP rs9387478, a lung cancer-associated SNP discovered by GWAS. We conducted a LD-based search of the HapMap and 1000Genomes database for SNPs that were located within 100 kb up and downstream of rs9387478 in Han Chinese using the Haploview softerware 4.2. Relevant SNPs were selected on the basis of following criteria: (i) in high LD with rs9387478 (*D*′ > 0.8); (ii) located in the coding or regulatory regions of the gene *DCBLD1*; (iii) were labeled as Category 1 by RegulomeDB (http://www.regulomedb.org/) [23, 24]; and (iv) had MAF > 0.05. Four SNPs in *DCBLD1* met these selection criteria, including rs17079281 in the promoter region, and rs6911915, rs9320604 and rs4946259 in the first intron. Detailed information on SNP genotyping was provided in Supplementary Methods.

### Cell culture

Human NSCLC cell lines A549 and NCI-H1299, embryonic kidney cell line HEK293T and lung normal epithelial cell lines Beas2B selected for in vitro experiments were purchased from the Shanghai Cell Bank, Type Culture Collection Committee of Chinese Academy of Science (CAS, Shanghai, China) and were authenticated using short tandem repeat profiling by the cell bank. These cell lines were passaged for fewer than 6 months. All cell lines were tested and confirmed to be negative for mycoplasma contamination. These cells were cultured in DMEM medium, containing 10% of fetal bovine serum. All the cell lines were incubated at 37^o^C with 5% CO_2_.

### Luciferase reporter assays

Detailed information on constructions of luciferase reporter gene plasmids was provided in Supplementary Methods. A549, NCI-H1299, and HEK293T were seeded at 5 × 10^4^ per well in 24-well plates and allowed to attach for 24 h. The cells were transfected with 0.4 μg of each reporter constructs (pGL3-Basic, pGL3-CC, and pGL3-TT) and 0.4 μg of either GV144-YY1 expression plasmid or empty GV144 vector using Lipofectamine 3000 (Invitrogen, USA) according to the manufacturer’s instructions. PRL-TK (8 ng) (Promega, USA) containing *Renilla* luciferase gene was co-transfected to standardize transfection efficiency. The relative luciferase activity was determined at 24 h after transfection using the Dual Luciferase Reporter Assay System (Promega, USA). The experiments were performed in triplicate experiments.

### CRISPR-Cas9 plasmid construction and establishment of knock-in clones

A 20-nt single-guide RNA (sgRNA) was designed based on the genomic sequences flanking rs17079281 and evaluated for potential off-target activity using the CRISPR Design tool at (http://crispr.mit.edu/). The sequence of sgRNA is as follows: sgRNA-top 5′-CACCgTCTCAGCTTTGATGCCACTT-3′ and sgRNA-bottom 5′-AAACAAGTGGCATCAAAGCTGAGA. sgRNA oligonucleotides were commercially synthesized (Sangon Biotech, China) and subcloned into *Bbs I* site of the pSpCas9(BB)-2A-Puro(px459) vector (a gift from Feng Zhang, Addgene plasmid #62988) according the manufacturer’s protocol [25]. The recombined plasmid (sgRNA-px459) was verified by Sanger sequencing. To generate the cells with homozygous T/T genotype of rs17079281, single-stranded DNA oligonucleotides (ssODNs) with flanking sequences of 50 bp on each side that are homologous to the target region was designed and synthesized. The sequence of ssODNs is 5′-GCACAACTTGTCTGTTTTGGCTCCTGCTTTAATCTCAGCTTTGATGCC

A**T**TTAGGCCTTTCCTAGCTGATTCCCGCCCTCACCCCTGTTATTACCCGCCATC-3′. Detailed information was provided in Supplementary Methods.

### Chromatin immunoprecipitation (CHIP) assay

We performed a CHIP analysis on the wild type Beas2B cells (C/T at rs17079281) and the modified Beas2B cells which had a knockin genotype (C/C at rs17079281) established using the CRISPR-cas9 technique. The Pierce^TM^ magnetic CHIP kit (Thermo Fisher Scientific, USA) was used for the CHIP assay according to the manufacturer’s instructions. About 20 μg chromatin was immunoprecipitated with 2 μg anti-YY1 antibody (Abcam, ab109237). Normal Rabbit IgG was used as control. An aliquot (1 μl) of each precipitated sample was further analyzed by qPCR analysis using the SYBR Green Mix (Life Technologies,USA). The primers used for qPCR were as follows, for the positive region: forward, 5′-TAAAAGCTAGCACTGCAACCTGG-3′ and reverse, 5′-TACTTAGTTTTGTCCCGT CAAAGAA-3′; for the target region: forward, 5′-TTGTCTGTTTTGGCTCCTGC-3′ and reverse 5′-ATTAGATGGCGGGTAACAGGG-3′. The CHIP results were normalized for chromatin input, control, and a positive region according to the following formulas: fold change over input = 2^(ct_input-ct_chip)^; negative norm = fold change over input_YY1_/fold change over input_IgG_; positive region norm = negative norm/negative norm_positive_.

### Analysis of cell proliferation, cell cycle, and colony formation

Detailed information on Lentivirus production and establishment of stable cell lines was provided in Supplementary Methods. Cell functional experiments were performed in lung cancer cell lines A549 and NCI-H1299.These assays were described in Supplementary Methods.

### Lung cancer cell line xenografts in mice

SPF grade BALB/c nude mice (male and female), aged 4–5 weeks, were purchased from Shanghai SLAC Laboratory Animal Co. Ltd. A549 cells stably transfected with shDCBLD1 and shControl lentiviral plasmids were respectively injected into the right and left blank flank of mice (*n* = 6) with 0.2 ml cell suspension mixture containing 0.1 ml PBS with 3 × 10^6^ cells and 0.1 ml matrigel. After 7 days, tumor size was measured every other day in non-blind way and tumor volume was calculated according to the formula: volume = length × width^2^ × 0.5. Portion of each xenograft tumor was fixed in 4% formaldehyde and embedded in paraffin for histological hematoxylin–eson (HE) staining. In addition, the tissue sections from paraffin-embedded xenograft tumor were stained with antibodies against Ki67(CST, #9449) and the IHC staining was quantified as previously described [9]. Protein and mRNA were extracted from xenograft tumor tissues to measure gene expression. The animal experiment was approved by the Shanghai Jiao Tong University School of Medicine Animal Ethics Committee and carried out in accordance to the approved protocols.

### Western blot and antibodies

Cells were lysed for 30 min on ice in 1× RIPA lysis buffer supplemented with a protease inhibitor cocktail and phosphatase inhibitors (Millipore, German). Denatured proteins (40 μg) were electrophoresed on 10% SDS-PAGE gel and were electrotransferred from the gel to PDVF membranes (Millipore, German). After blocking with 5% no-fat milk in PBS with 0.05% Tween-20 for 1 h at room temperature, the membranes were incubated overnight at 4 °C with primary antibodies against DCBLD1 (Abcam, ab185216), cyclin D1 (Abcam, ab134175), cyclin E1 (CST, 4129S), anti-YY1 antibody (Abcam, ab109237), β-actin at 1:1000 (Sigma-Aldrich, A1978). IRDye 800CW secondary antibodies were added at concentrations of 1:4000. LI-COR odyssey system was used to visualize protein expression.

### RNA extraction and qRT-PCR analysis

These assays were performed as described in Supplementary Methods. Primer sequences are detailed in Supplementary Table S4.

### Statistical analysis

Chi-square test was used to compare the differences in distributions of demographic variables, risk factors, and SNP genotypes between cases and controls. Hardy–Weinberg equilibrium was calculated in the control subjects using the goodness-of-fit chi-square test to compare the expected genotypes with the observed ones. To estimate the associations between SNPs and lung cancer risk in additive, dominate, and recessive models, unconditional logistic regression models were used with adjustment for age, gender, smoking status, BMI, and family history of cancer. Odds ratios (ORs) and 95% confidence intervals (CIs) were calculated in the model. *P* value for trend was defined as the genotypes in a regression model as a continuous ordinal variable [26]. SAS software version 9.1 (SAS, US) was used for statistical analysis. Cell experiment data were analyzed using GraphPad Prism 6 software (GraphPad). One-way ANOVA was used for 3-group comparison. LSD*-t* multiple comparison test was performed for intergroup comparisons. Student’s *t* test was applied for 2-group comparison. Data were presented as mean ± SEM. All statistical tests were two-sided, and *P* value ≤ 0.05 was selected as the significant level.

## Supplementary information

supplementary methods

supplementary table S1-4

supplementary figure1

Supplementary legends
