# Supplementary material for: SNP rs17079281 decreases lung cancer risk through creating an YY1-binding site to suppress DCBLD1 expression
Source: Oncogene. 2020 Mar 30;39(20):4092–102. doi: 10.1038/s41388-020-1278-4 (PMC7220863; doi:10.1038/s41388-020-1278-4)
Supplement: Supplementary file 1 — supplementary methods [file 41388_2020_1278_MOESM1_ESM.docx]

**Supplementary Methods**

**Study subjects**

All patients in the study were diagnosed with histologically confirmed primary non-small cell lung cancer (NSCLC). Patients with a previous history of cancer or radio/chemotherapy were excluded from the study. Patient information on tumor histology and body weight and height were extracted from their medical charts and pathology reports. Healthy individuals who underwent regular health checkup during the same time as the patients being recruited were enrolled in the study as controls. The control subjects were matched to cases on gender and age (±5 years). In addition, Tumor specimens were collected from 170 lung cancer patients in Tianjin Medical University Cancer Hospital.

All study participants were unrelated Han ethnic Chinese and were interviewed by trained research staff using a structured questionnaire which ascertained information on demographic features, medical history of lung diseases, family history of cancer, status of tobacco smoking, years of smoking, and number of cigarettes smoked per day. Individuals who had smoked at least one cigarette per day for more than 12 months in lifetime were defined as smokers. Pack-years was calculated by multiplying the number of packs smoked daily with the number of years smoked. Family history of cancer was defined as self-reported cancer in the first-degree relatives.

**SNP genotyping**

Genomic DNA was extracted from peripheral blood using the TKM method and genotyped with the TaqMan assays using the 7900HT Fast Real-time PCR System. The TaqMan assays targeting on the 4 SNPs were designed and manufactured by ABI (Applied Biosystems Inc., USA), and the PCR assays were performed according to the manufacturer’s instructions. Five percent of the samples were randomly selected for retesting, and the results were in complete concordance.

**Constructions of luciferase reporter gene plasmids**

Our bioinformatic analysis, based on TRANSFAC® (http://www.gene- regulation.com/pub/databases.html) and ALGGEN PROMO (<http://alggen>. lsi.upc.es/cgi-bin/promo_v3/promo/promoinit.cgi/DirDB=TF_8.3), indicated that SNP rs17079281, a C/T polymorphism, might affect the binding affinity of the transcription factor YY1 to the *DCBLD1* promoter (Fig 1A). To determine whether this polymorphism has an impact on the transcriptional activity of *DCBLD1*, we constructed two luciferase reporter plasmids containing either C or T allele of rs17079281. DNA fragments with 1070 bp bearing the *DCBLD1* promoter region (nucleotides -959 to +110，relative to the transcription start site) were amplified by PCR using the genomic DNA isolated from control subjects who carry either homozygous C/C or T/T genotype. The PCR primers were designed as follows: forward with *KPN I* site, 5’-GGGGTACCCCTCCCCAAACCCTTCTCCGC-3’ and reverse with *Bgl II* site, 5’-GAAGATCTCAGCTTGGCAAGCTCGGCCT-3’. PCR conditions included initial denaturing at 95^o^C for 10 min and 35 cycles at 94^o^C for 40 secs, 64.9^o^C for 30 secs and 72^o^C for 1 min and 20 secs, followed by elongation at 72^o^C for 10 min. The PCR products between the *KPN I* and *Bgl II* sites were cloned into the pGL3-Basic vector (Promega, USA) containing the firefly luciferase gene as a reporter. The recombined vector containing the C or T allele of rs17079281 were confirmed by direct sequencing. The resultant plasmid containing C allele of rs17079281 was designated as pGL3-CC; the resultant plasmid with T allele was named pGL3-TT. To elucidate the effect of transcription factor YY1 on transactivation, an YY1 overexpression vector (GV144-YY1) containing a GFP tag in N term and its negative control were purchased from Genechem (Shanghai, China).

**CRISPR-Cas9 plasmid construction and establishment of knock-in clones**

HEK293T cells were seeded into 6-well plate and transfected with 2.5μg of the sgRNA-px459 plasmid using Lipofectamine 3000 (Thermo Fisher Scientific, USA). After 24 hours of transfection, cells which survived 1μg puromycin (Sigma, UK) for 48 hours were selected. The genomic DNA was isolated and PCR amplification of a region surrounding the targeted site was carried out to examine the efficiency of sgRNA cleavage. Beas2B (C/T at rs17079281) cells were seeded at a density 3×10^5^ cells per well for 24 hours with co-transfected 2.5μg sgRNA plasmid and 10μM ssODNs. Cells were selected after incubation with 1μg puromycin for 48 hours. The selected cells were dissociated into single cell with Tyrisin and transferred into 15 cm plate. Single cells were expanded for 14 days to form colonies, and the colonies were picked to place in 48 well plates. When these cells reached 70%-80% confluence, genomic DNA was extracted using the QuickExtract^TM^ DNA Extraction Solution (Epicenter, Sweden). PCR amplified the region surrounding the SNP site. The PCR conditions included: 94℃for 2 min and 33 cycles at 94℃ for 30 sec, 64.8℃ for 30 sec and 68℃ for 1 min and 20 sec, followed by elongation at 68℃ for 5 min. The PCR primers were: forward, 5’-CGCAGAGGAAGTAAACGGGGACTA-3’ and reverse,5’-CAC

CAGTACACGTGAACCTGCTAG-3’. The homozygous genotype clones were confirmed by Sanger sequencing of the PCR products.

**Plasmids transfection**

The wild type Bease2B (C/T at rs17079281) and the modified Beas2B cells (C/C at rs17079281) were seeded at 3×10^6^ concentrations per well in 6-well plates. The next day, these cells were transfected with 1 μg or 2 μg of YY1 overexpression plasmid and its negative control using the ploy Jet (SignaGen Laboratories,USA). After incubation for 48 hours’ post-transfection, the expression of the targeted genes at mRNA and protein levels were analyzed using qRT-PCR and western blot, respectively.

**Lentivirus production and establishment of stable cell lines**

Lentiviral shRNA plasmid targeting *DCBLD1* (shDCBLD1) and negative control plasmid (shcontrol) expression GFP were generated by Genechem Company. The target sequences for the shRNAs were as follows: DCBLD1 5’-TGAGTTTACCATCAGCTAT-3’, control 5’-TTCTCCGAACGTGTCACGT-3’. Lentiviral plasmid with DCBLD1 overexpression pCDH-EF1 -DCBLD1 and pCDH-EF1 empty vector were purchased from IBS Company (China). All lentiviruses were generated by transient co-transfection of HEK293T cells using PolyJet (SignaGen Laboratories, USA) with a three-plasmid combination including lentiviral shRNA plasmid or overexpression plasmid (5 μg), PMD2.G (1.25 μg) and psPAX2 (3.75 μg). Supernatants were collected at 48 and 72 h post transfection, filtered through 0.45-μm PVDF filters and frozen at -80 ºC. A549 cells were seeded at 3×10^6^ in 10 cm dish and transfected with 4 ml recombinant lentiviruses in the presence of 5 μg of polybrene per ml. The supernatant was replaced with DMEM medium, containing 10% FBS after 24 hours, followed by puromycin selection (2 μg/ml). After incubation for three days, we observed green fluorescence and the expression of DCBLD1 was verified by qRT-PCR and western blot

**Analysis of cell proliferation, cell cycle, colony formation, invasion and migration**

For CCK8 assay, cells were seeded in 96-well plate with each well containing 3000 cells in 100 μl of cell suspension. After 24, 48, 72 and 96 hours, cell viability was measured using the CCK8 assay (Dojindo, Japan). For cell cycle assay, cells were collected and fixed in 70% ethanol overnight at -20℃.The fixed cells were washed in PBS and resuspended in PBS containing both 0.1mg/ml RNase (Sigma, Irvine, UK) and 0.1mg/ml propidium iodide (Sigma, Irvine, UK), incubated at 37 for 30min. The cell solutions were analyzed by a flowcytometer, BD C6 ™. The results were analyzed with FlowJo software (BD Bioscience, USA). For colony formation assay, 200 cells were seeded in 10 cm dishes and grew until visible colonies in complete growth medium (14 days). The colonies were fixed with 4% paraformaldehyde and stained with crystal violet (Sangon Biotech, China). Each experiment was carried out in six replicates and repeated three times.

**RNA extraction and qRT-PCR analysis**

Total RNA was extracted from lung tumor samples using the TRizol reagents. RNA (5ug) was then reverse transcribed to complementary DNA using oligo (dT) and MLV (Invitrogen, US). The cDNA level was relatively quantified in the 7900HT Fast Real-time PCR system using the TaqMan gene expression assay according to the manufacturer’s protocol. Levels of β-actin expression were measured as internal reference, and each sample was tested in triplicate. Log(2^-ΔΔCt^) was calculated as the level of mRNA expression.

Total RNA was extracted from the cell lines and xenograft tumor tissues using the TRizol reagents. RNA (5 μg) was then reverse transcribed to complementary DNA using High Capacity cDNA Reverse Transcription Kit (Thermo Fisher Scientific, USA). The cDNA level was relatively quantified in the 7900HT Fast Real-time PCR system using the SYRB Green method according to the manufacturer’s protocol. The results were normalized using β-actin and fold-change values were calculated based on the 2^-ΔΔct^ method, and each sample was tested in triplicate. Primer sequences are detailed in Supplemental_Table_S1.

**DCBLD1 expression in lung cancer tissue**

We analyzed the *DCBLD1* expression in lung cancer tissues and matched adjacent normal tissue from two publically available datasets, GEO and TCGA. The dataset GSE19804 had 60 pairs of tumor and adjacent normal lung tissues from Chinese nonsmoking female patients with non-small cell lung carcinoma (NSCLC) in Taiwan. The gene expression data were generated from the Affymetrix Human Genome U133 Plus 2.0 Array. We also found a TCGA dataset which contained *DCBLD1* expression data in matched tumors and adjacent normal tissues of 108 lung cancer tissues.
