# Supplementary material for: SNP rs17079281 decreases lung cancer risk through creating an YY1-binding site to suppress DCBLD1 expression
Source: Oncogene. 2020 Mar 30;39(20):4092–102. doi: 10.1038/s41388-020-1278-4 (PMC7220863; doi:10.1038/s41388-020-1278-4)
Supplement: Supplementary file 2 — supplementary table S1-4 [file 41388_2020_1278_MOESM2_ESM.docx]

**Table S1. Characteristics of lung cancer patients and healthy controls**

| **Variables** | **N（%）** | | ***P^a^*** | ***OR*（95%*CI*）** |
| --- | --- | --- | --- | --- |
|  | **Control（773）** | **Case（766）** |  |  |
| **Age at diagnosis** |  |  | 0.350 |  |
| <60 | 404(52.26) | 382(49.87) |  | 1.00 |
| ≥60 | 369(47.74) | 384(50.13) |  | 1.05（0.94-1.16） |
| **Gender** |  |  | 0.510 |  |
| Male | 466（60.28) | 468(61.90) |  | 1.00 |
| Female | 307(39.72) | 288(38.10) |  | 0.96(0.87-1.07) |
| **Lung disease history** |  |  | 0.710 |  |
| No | 550(90.02) | 685(90.61) |  | 1.00 |
| Yes | 61(9.98) | 71（9.39） |  | 0.90（0.67-1.30） |
| **Family history of cancer** |  |  | 0.072 |  |
| No | 671(87.83) | 634(84.65) |  | 1.00 |
| Yes | 93(12.17) | 115(15.35) |  | 1.26(0.97-1.62) |
| **Smoking status** |  |  | **<0.001** |  |
| No | 475(61.45) | 269(35.77) |  | 1.00 |
| Yes | 298(38.55) | 483(64.23) |  | **1.66(1.15-1.84)** |
| **Pack-years** |  |  | **<0.001** |  |
| 0 | 475(66.43) | 269(36.30) |  | 1.00 |
| 0.50-35 | 145(20.28) | 203(27.40) |  | **2.40(1.90-3.20)** |
| ≥35 | 95(13.29) | 269(36.30) |  | **5.00(3.70-6.60)** |
| **BMI** |  |  | **<0.001** |  |
| >24 | 227(34.87) | 348(47.22) |  | 1.00 |
| 24-28 | 264(40.55) | 295(40.03) |  | **0.73(0.57-0.92)** |
| >28 | 160(24.58) | 94(12.75) |  | **0.38(0.28-0.52)** |

^a^ *P* values for a two-sidedχ^2^test

**Table S2. SNPs in 6q22.2 that are in high LD with rs9387478, based on the genotype information in the CHB population in the HapMap and 1000 Genomes**

| **SNPs** | **Chr.** | **Position** | **location** | **D’** | **r^2^** | **MAF** | **RegulomeDB score** |
| --- | --- | --- | --- | --- | --- | --- | --- |
| rs6937083 | 6 | 117785308 | NA | 1 | 1 | 0.48 | 5 |
| rs34882116 | 6 | 117789497 | NA | 1 | 1 | 0.48 | 6 |
| rs9374663 | 6 | 117782634 | NA | 1 | 1 | 0.48 | No data |
| rs4946256 | 6 | 117781276 | NA | 1 | 1 | 0.47 | 6 |
| rs2180811 | 6 | 117780158 | NA | 1 | 1 | 0.47 | 6 |
| rs9374662 | 6 | 117777804 | NA | 1 | 1 | 0.47 | No data |
| rs9372480 | 6 | 117760579 | NA | 0.96 | 0.89 | 0.47 | No data |
| rs4945584 | 6 | 117750980 | NA | 0.96 | 0.89 | 0.5 | 6 |
| rs1853148 | 6 | 117750980 | NA | 0.96 | 0.89 | 0.49 | 6 |
| rs6941337 | 6 | 117768448 | NA | 1 | 0.87 | 0.45 | 6 |
| rs2883418 | 6 | 117791751 | NA | 1 | 0.86 | 0.43 | No data |
| rs9387479 | 6 | 117792612 | NA | 1 | 0.86 | NA | No data |
| rs6940922 | 6 | 117798204 | NA | 1 | 0.86 | 0.43 | 5 |
| rs2354346 | 6 | 117798509 | NA | 1 | 0.86 | 0.43 | 4 |
| rs4946254 | 6 | 117765313 | NA | 0.98 | 0.82 | 0.43 | 6 |
| rs9387481 | 6 | 117810822 | DCBLD1  intron3 | 0.98 | 0.82 | NA | 6 |
| rs9489213 | 6 | 117811706 | DCBLD1  intron3 | 0.98 | 0.80 | 0.45 | 6 |
| rs12527127 | 6 | 117813269 | DCBLD1  intron3 | 0.98 | 0.80 | 0.45 | 4 |
| rs4945586 | 6 | 117815020 | DCBLD1  intron3 | 0.98 | 0.80 | 0.45 | 4 |
| rs9481728 | 6 | 117817165 | DCBLD1  Intron4 | 0.98 | 0.80 | 0.45 | 2b |
| rs929057 | 6 | 117818911 | DCBLD1  Intron4 | 0.98 | 0.80 | 0.45 | 5 |
| rs2057314 | 6 | 117818911 | DCBLD1  Intron4 | 0.98 | 0.80 | 0.44 | 4 |
| rs9688361 | 6 | 117821888 | DCBLD1  Intron4 | 0.98 | 0.80 | 0.44 | 6 |

| rs4946260 | 6 | 117822993 | DCBLD1  Intron4 | 0.98 | 0.80 | 0.45 | 4 |
| --- | --- | --- | --- | --- | --- | --- | --- |
| rs10782186 | 6 | 117823508 | DCBLD1  Intron4 | 0.98 | 0.80 | 0.50 | No data |
| rs1555401 | 6 | 117767335 | NA | 0.81 | 0.49 | 0.35 | 1f |
| rs9489193 | 6 | 117769728 | NA | 0.86 | 0.41 | 0.3 | 5 |
| rs6930292 | 6 | 117783770 | NA | 0.88 | 0.5 | 0.4 | No data |
| rs717969 | 6 | 117784567 | NA | 0.88 | 0.48 | 0.38 | 6 |
| rs6942067 | 6 | 117785696 | NA | 0.88 | 0.48 | 0.33 | 4 |
| rs7749229 | 6 | 117787897 | NA | 0.87 | 0.42 | 0.35 | 5 |
| rs13205986 | 6 | 117791674 | NA | 0.87 | 0.46 | 0.37 | 6 |
| rs2104064 | 6 | 117793798 | NA | 0.86 | 0.4 | 0.34 | 1f |
| rs7763979 | 6 | 117795970 | NA | 0.87 | 0.46 | 0.37 | 6 |
| rs7746536 | 6 | 117799572 | NA | 0.87 | 0.46 | 0.37 | 4 |
| **rs17079281** | **6** | **117803138** | **DCBLD1 5’flanking** | **0.86** | **0.4** | **0.34** | **1b** |
| **rs6911915** | **6** | **117809031** | **DCBLD1 intron1** | **0.93** | **0.83** | **0.5** | **1f** |
| **rs9320604** | **6** | **117816045** | **DCBLD1 intron1** | **0.88** | **0.78** | **0.45** | **1f** |
| **rs4946259** | **6** | **1177816093** | **DCBLD1 intron1** | **0.86** | **0.68** | **0.4** | **1f** |

**Table S3. Associations between lung cancer and SNP rs17079281 in a validation set**

| **Genotype** | **N (%)** | | ***P^a^*** | ***OR (95%CI)^b^*** |
| --- | --- | --- | --- | --- |
|  | **Control (534)** | **Case (558)** |  |  |
| ***DCBLD1* rs17079281(C>T)** |  |  | **0.023** |  |
| CC | 228(42.70) | 272(48.70) |  | 1 |
| CT | 261(48.70) | 228(40.90) |  | **0.73(0.56-0.95)** |
| TT | 45(8.40) | 58(10.40) |  | 1.16(0.74-1.83) |
| **P trend** |  |  | 0.396 |  |
| **Dominant model** |  |  |  |  |
| CC | 228(42.70) | 272(45.80) |  | 1 |
| CT+TT | 306(57.30) | 286(54.20) |  | **0.79(0.62-1.00)** |
| **Recessive model** |  |  |  |  |
| CC+CT | 489(91.60) | 500(89.60) |  | 1 |
| TT | 45(8.40) | 58(10.40) |  | 1.35(0.88-2.09) |

a Two-side, and calculated by logistic regression analysis

b Adjusted by age, gender, smoke, BMI

**Table S4. The primers used in the qRT-PCR method**

| Gene name | Sequence (5'-3') |
| --- | --- |
| *DCBLD1*-F | ACACAAGTGAAGTAACCGTCCG |
| *DCBLD1*-R | TGCTACGTCTCTACAACCAGC |
| *CCND1*-F | GCTGCGAAGTGGAAACCATC |
| *CCND1*-R | CCTCCTTCTGCACACATTTGAA |
| *CCNE1*-F | GCCAGCCTTGGGACAATAATG |
| *CCNE1*-R | CTTGCACGTTGAGTTTGGGT |
| *YY1*-F | CCTCTCAGATCCCAAACAACTG |
| *YY1*-R | GCCTTTATGAGGGCAAGCTATT |
