# Supplementary figures and images for: SNP rs17079281 decreases lung cancer risk through creating an YY1-binding site to suppress DCBLD1 expression
Source: Oncogene. 2020 Mar 30;39(20):4092–102. doi: 10.1038/s41388-020-1278-4 (PMC7220863; doi:10.1038/s41388-020-1278-4)

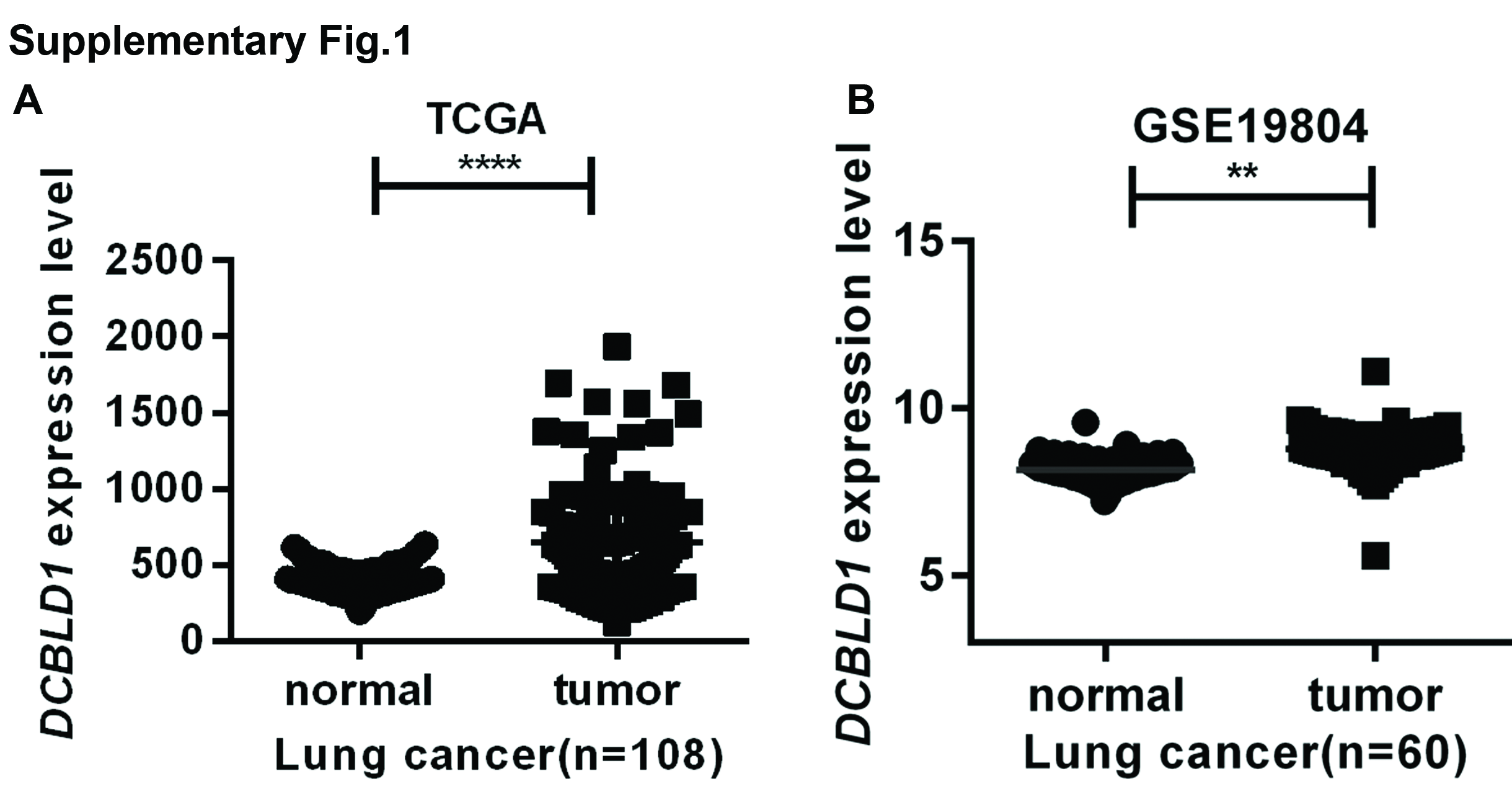

Supplement: Supplementary file 3 — supplementary figure1 [file 41388_2020_1278_MOESM3_ESM.tif]
