# Supplementary material for: SNP rs17079281 decreases lung cancer risk through creating an YY1-binding site to suppress DCBLD1 expression
Source: Oncogene. 2020 Mar 30;39(20):4092–102. doi: 10.1038/s41388-020-1278-4 (PMC7220863; doi:10.1038/s41388-020-1278-4)
Supplement: Supplementary file 4 — Supplementary legends [file 41388_2020_1278_MOESM4_ESM.docx]

**Supplementary Fig.1.** **DCBLD1 expression in lung cancer tissue** (A) DCBLD1 expression in tumor and adjacent normal tissues of 108 pairs lung tissues from the TCGA database. Results were shown as means±SEM. *****P*<0.001, all *P* values were from paired *t* tests. (B) Expression of DCBLD1 in 60 pairs of lung cancer tissues and adjacent normal tissues from the GEO database (GSE19804). Results were shown as means±SEM.***P*<0.01, all *P* values were from paired *t* tests.
